# Association between triglyceride glucose-waist height ratio index and cardiovascular disease in middle-aged and older Chinese individuals: a nationwide cohort study

**DOI:** 10.1186/s12933-024-02336-6

**Published:** 2024-07-11

**Authors:** Qiushi Ren, Yang Huang, Quan Liu, Tongxin Chu, Gang Li, Zhongkai Wu

**Affiliations:** 1 Department of Cardiac Surgery, First Affiliated Hospital of Sun Yat-Sen University, 58 Zhongshan II Road, Guangzhou, 510080 China; 2 NHC Key Laboratory of Assisted Circulation, Sun Yat-Sen University, Guangzhou, China

**Keywords:** Cardiovascular disease, Triglyceride glucose-waist height ratio, K-means clustering, CHARLS

## Abstract

**Background:**

The triglyceride-glucose (TyG) index and its combination with obesity indicators can predict cardiovascular diseases (CVD). However, there is limited research on the relationship between changes in the triglyceride glucose-waist height ratio (TyG-WHtR) and CVD. Our study aims to investigate the relationship between the change in the TyG-WHtR and the risk of CVD.

**Methods:**

Participants were from the China Health and Retirement Longitudinal Study (CHARLS). CVD was defined as self-reporting heart disease and stroke. Participants were divided into three groups based on changes in TyG-WHtR using K-means cluster analysis. Multivariable binary logistic regression analysis was used to examine the association between different groups (based on the change of TyG-WHtR) and CVD. A restricted cubic spline (RCS) regression model was used to explore the potential nonlinear association of the cumulative TyG-WHtR and CVD events.

**Results:**

During follow-up between 2015 and 2020, 623 (18.8%) of 3312 participants developed CVD. After adjusting for various potential confounders, compared to the participants with consistently low and stable TyG-WHtR, the risk of CVD was significantly higher in participants with moderate and increasing TyG-WHtR (OR 1.28, 95%CI 1.01–1.63) and participants with high TyG-WHtR with a slowly increasing trend (OR 1.58, 95%CI 1.16–2.15). Higher levels of cumulative TyG-WHtR were independently associated with a higher risk of CVD events (per SD, OR 1.27, 95%CI 1.12–1.43).

**Conclusions:**

For middle-aged and older adults, changes in the TyG-WHtR are independently associated with the risk of CVD. Maintaining a favorable TyG index, effective weight management, and a reasonable waist circumference contribute to preventing CVD.

**Supplementary Information:**

The online version contains supplementary material available at 10.1186/s12933-024-02336-6.

## Introduction

Cardiovascular diseases (CVD) are the leading cause of global mortality. Although global age-standardized CVD mortality decreased by 34.9% from 1990 to 2022, the actual number of CVD deaths has increased significantly [1, 2]. 2022 alone, CVD caused an estimated 19.8 million deaths worldwide, corresponding to 396 million years of life lost and another 44.9 million years lived with disability (YLD) [1, 2]. Early identification of high-risk groups for CVD and timely intervention to control CVD risk factors contribute to preventing the disease progression.

Insulin resistance (IR) is associated with asymptomatic atherosclerosis and coronary artery disease and is considered as predictor of CVD [3, 4]. The triglyceride-glucose index has high sensitivity and specificity in assessing IR in individuals [5]. Recent studies showed that, as a valuable biomarker of IR, triglyceride-glucose index(TyG) could be used to predict the occurrence of CVD [6, 7]. Furthermore, TyG also performed excellently in predicting the outcomes of CVD patients [8, 9].

Obesity is closely associated with various health risks, and increased abdominal fat is linked to CVD via multiple direct and indirect pathophysiological mechanisms [10]. Some studies proved that TyG combined with adiposity indices performs better than only the TyG index in assessing IR and cardiovascular risk [11]. Two previous studies used the TyG index at baseline combined with different obesity indicators to form new indicators, including TyG-BMI, TyG-WC, and TyG-WhtR, to analyze the association of these new indicators and CVD events [11, 13]. These studies were from the National Health and Nutrition Examination Survey (NHANES) [11] and the Kailuan study [13]. TyG-WHtR performed the best in identifying high-risk populations for CVD events and CVD mortality. WHtR is a standardized waist circumference measurement method that is more objective than simple waist circumference measurement. Previous studies were primarily based on the TyG index and related indicators at a single time point. However, the TyG index is a dynamic state that changes over time. We hypothesized that the dynamic change of TyG-WHtR can better predict CVD. Our study aims to investigate the relationship between the change of TyG-WHtR and the risk of CVD.

## Methods

### Study design and population

Our study is based on data from the China Health and Retirement Longitudinal Study (CHARLS), a national population-based cohort study. The study design has been previously described [14]. The CHARLS national baseline survey (Wave 1) spanned from June 2011 to March 2012, encompassing 17,708 individual participants. A multistage probability sampling technique selected participants from 150 counties or regions and 450 villages or urban communities in China. Up to date, four subsequent follow-up surveys were conducted every 2 years (Wave 2 in 2013, Wave 3 in 2015, Wave 4 in 2018, and Wave 5 in 2020).

**Fig. 1:**
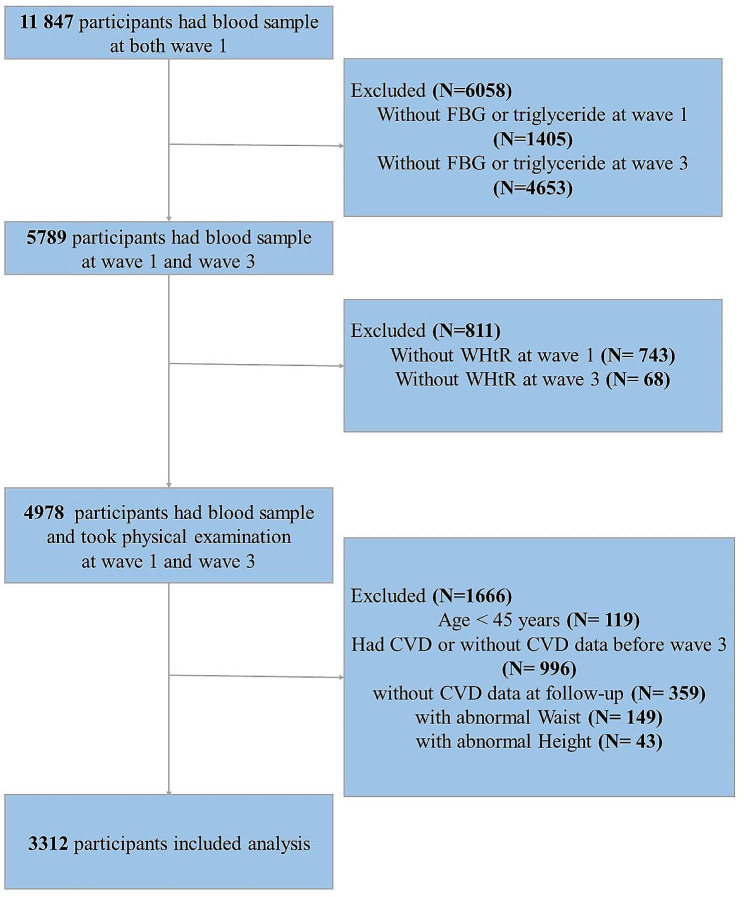
Flow chart of the study population

In this study, we included 11,847 participants who participated in blood tests. We excluded participants for reasons including (1) lack of fasting blood glucose (FBG) or triglycerides (TG) at Wave 1 or Wave 3. (2) Missing or abnormal height and waist measurements. (3) under 45 years of age. (4) People who had CVD events before Wave 3 (2015) or lacked information about CVD. The detailed inclusion and exclusion process is shown in Fig. 1.

The Biomedical Ethics Review Board of Peking University gave its ethical approval for gathering CHARLS data (IRB00001052-11015), and all participants signed an informed consent form.

### Assessment of the change in TyG-WHtR

Fasting status, blood glucose, and triglyceride levels were obtained from the blood sample examinations at Wave 1 and Wave 3. Any height variation exceeding 10 cm between measurements taken at Wave 1 and Wave 3 was deemed an abnormal height measurement. Any waist measurement that deviates from the overall mean waist measurement by more than three standard deviations (either above or below) was classified as an abnormal waist measurement. TyG, WHtR, TyG-WHtR, and cumulative TyG-WHtR were calculated according to the following formulas [11, 15]: (1) TyG = ln [triglycerides (mg/dl) × glucose (mg/dl)/2]; (2) WHtR = waist circumference/height; (3) TyG-WHtR = TyG × WHtR; (3) Cumulative TyG-WHtR = (TyG-WHtR2012 + TyG-WHtR2015)/2 × time(2015–2012).

### Assessment of CVD

The presence of heart disease was determined by the question, “Have you been diagnosed with heart attack, coronary heart disease, angina, congestive heart failure, or other heart problems by a doctor?” Similarly, the occurrence of stroke was ascertained through the question, “Have you been diagnosed with stroke by a doctor?” CVD was defined as self-reporting heart disease and stroke. Participants were required to reaffirm CVD in the subsequent wave if they reported heart disease or stroke in the previous wave. When participants denied their previous self-reported diagnoses of heart disease or stroke, these inconsistencies were rectified retrospectively. Our CVD ascertainment was consistent with previous studies using the CHARLS [16].

### Covariates

The covariates included sociodemographic characters (age, sex, marital status, hukou status, education, smoking status, and drinking status), health conditions (systolic blood pressure, diastolic blood pressure, hypertension, diabetes, dyslipidemia, and cancer), and laboratory examination total cholesterol (TC), high-density lipoprotein cholesterol (HDL-C), low-density lipoprotein cholesterol (LDL-C), and glycosylated hemoglobin (HbA1c)). Hypertension was defined as systolic blood pressure ≥ 140 mmHg or diastolic blood pressure ≥ 90 mmHg or self-reported diagnosis history of hypertension or use of any antihypertensive treatment. Diabetes was defined as fasting glucose ≥ 7.0 mmol/L or self-reported diagnosis history of diabetes or use of any hypoglycemic medication. Dyslipidemia was defined as TC ≥ 240 mg/dl, triglycerides ≥ 150 mg/dl, LDL-C ≥ 160 mg/dl, HDL-C < 40 mg/dl, self-reported dyslipidemia or use of lipid-lowering treatment [15].

### Statistical analyses

**Fig. 2:**
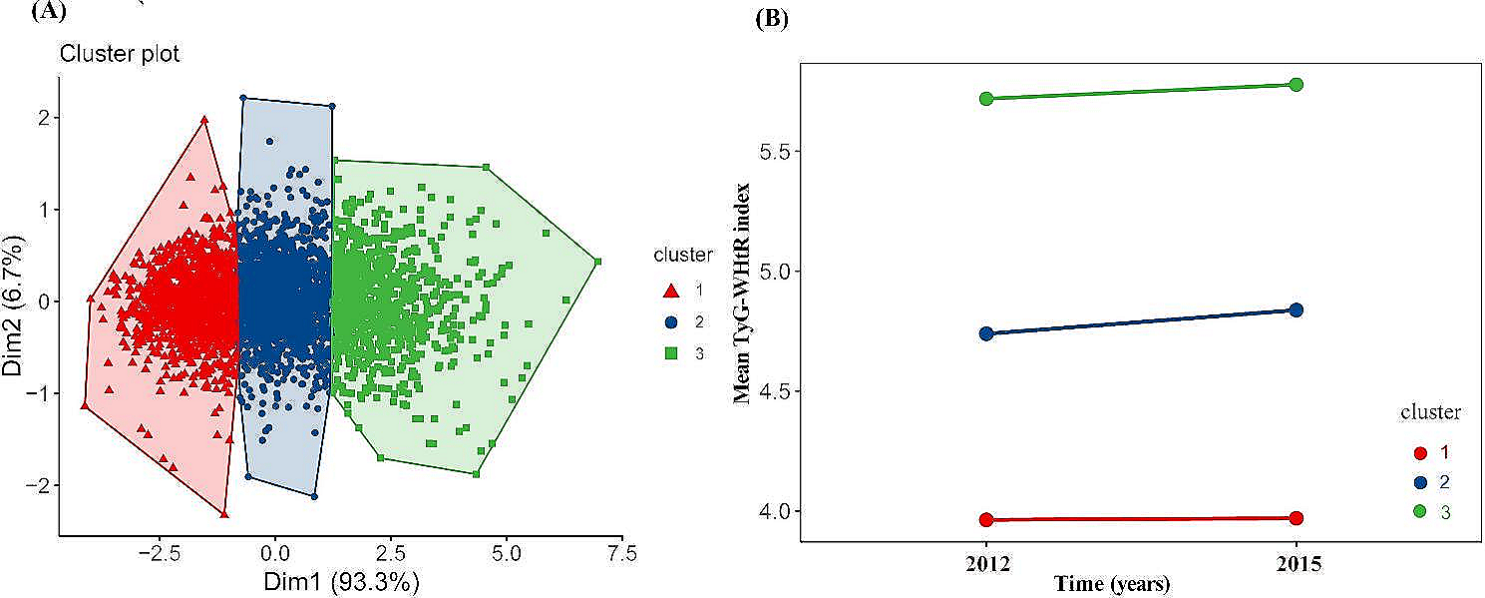
Clustering of the change in the TyG-WHtR from 2012 to 2015. **A** Three clusters were found using the K-means method with Euclidean distance: the x- and y-axes are principal components of the change in the TyG-WHtR; **B** The change of TyG-WHtR from 2012 to 2015

K-means clustering analysis is a type of unsupervised machine learning method. We use the elbow method to identify the appropriate number of clusters (Additional file 1: Figure S1). Based on measurements of TyG-WHtR in 2012 and 2015, We classify the participants into three groups: for Class 1 (n = 1141), the TyG-WHtR ranged from 3.96 ± 0.33 in 2012 to 3.97 ± 0.36 in 2015, and the cumulative TyG-WHtR was 11.90 ± 0.82, representing a consistently low and stable TyG-WHtR; for Class 2 (n = 1392), the TyG-WHtR ranged from 4.74 ± 0.34 in 2012 to 4.84 ± 0.34 in 2015, and the cumulative TyG-WHtR was 14.36 ± 0.72, representing a moderate and increasing TyG-WHtR; for Class 3 (n = 779), the TyG-WHtR ranged from 5.72 ± 0.49 in 2012 to 5.78 ± 0.47 in 2015, and the cumulative TyG-WHtR was 17.24 ± 1.18, representing a high TyG-WHtR with a slowly increasing trend (Fig. 2).

Continuous variables were described as mean (standard deviation, SD) or median (interquartile range, IQR), while categorical variables were expressed as frequency (proportion). We used multivariable binary logistic regression analysis to examine the association between different clusters (based on measurements of TyG-WHtR) and CVD. The results were shown as odds ratio(ORs) and 95% confidence intervals (CIs). Four models were estimated: Model 1 adjusted for age and gender. Model 2 adjusted for age, gender, marital status, hukou status, educational level, smoking status, and drinking status. Model 3 adjusted for variables in Model 2 and history of hypertension, diabetes, dyslipidemia, and cancer. Model 4 adjusted for variables in Model 3 and systolic blood pressure, diastolic blood pressure, total cholesterol, HDL-C, LDL-C, and HbA1c. We divided participants into four groups based on quartiles of cumulative TyG-WHtR to examine the relationship between cumulative TyG-WHtR and CVD. P values for trend were calculated using the median TyG-WHtR in each quartile. We also used a restricted cubic spline (RCS) regression model to explore the potential nonlinear association of the cumulative TyG-WHtR and CVD events. We fit restricted cubic spline models with 3–5 nodes and then selected the model with the smallest AIC to determine the number of nodes. Additionally, we performed subgroup and interaction analyses to investigate whether the relationships between the change in TyG-WHtR or cumulative TyG-WHtR and CVD varied according to the status of the covariates (gender, marital status, education level, smoking status, drinking status, hypertension, diabetes, and dyslipidemia). Receiver operating characteristic (ROC) curves were used for diagnostic value analysis, and the area under the curve was computed to quantify the predictive power of TyG, WHtR, TyG-WHtR, and cumulative TyG-WHtR for cardiovascular disease. Missing covariates were imputed by multiple imputations using mice package in R. We also repeated analyses using the complete data set (3216 participants) without multiple imputations as sensitivity analysis. A two-sided P value < 0.05 was considered statistically significant. Statistical analysis was performed using R software 4.3.3 and SPSS 26.0.

## Results

### Baseline characteristics of participants

In this study, 3312 participants were included for analysis. The mean age at baseline was 58.30 ± 8.38 years, and 1499(45.3%) were men. The mean TyG-WHtR was 4.70 ± 0.76 in 2012 and 4.76 ± 0.78 in 2015, and the mean cumulative TyG-WHtR was 14.19 ± 2.19. Based on the cluster analysis, the baseline characteristics of participants in each group are presented in Table 1. Additionally, we described the baseline characteristics of participants in each group based on the cumulative TyG-WHtR (Additional file 1: Table S1).

**Table 1:** Baseline characteristics of participants by K-means clustering analysis

Characteristic	Overall (n = 3312)	Change in the TyG-WHt			P value
		Class 1 (n = 1141)	Class 2 (n = 1392)	Class 3 (n = 779)	
Age,years	58.30 ± 8.38	58.02 ± 8.49	58.44 ± 8.41	58.60 ± 8.10	0.232
Gender^a^					< 0.001
Male	1499 (45.3)	738 (64.8)	564 (40.6)	197 (25.3)	
Female	1808 (54.7)	401 (35.2)	826(59.4)	581 (74.7)	
Marital status					0.943
Married	2981 (90.0)	1029 (90.2)	1250 (89.8)	702 (90.1)	
Others	331 (10.0)	112 (9.8)	142 (10.2)	77 (9.9)	
Education					0.001
Lower level	2304 (69.6)	770 (67.5)	951 (68.3)	583 (74.8)	
Higher level	1008 (30.4)	371 (32.5)	441 (31.7)	196 (25.2)	
Hukou^a^					< 0.001
Agriculture	2858 (86.3)	1028 (90.1)	1161 (83.5)	669 (85.9)	
Others	453 (13.7)	113 (9.9)	230 (16.5)	110 (14.1)	
Smoking status ^a^					< 0.001
Never	2068 (62.6)	524 (46.0)	938 (67.5)	606 (77.9)	
Previous	241 (7.3)	89 (7.8)	111 (8.0)	41 (5.3)	
Current	997 (30.2)	526 (46.2)	340 (24.5)	131 (16.8)	
Drinking status					< 0.001
Current	1125 (34.0)	502 (44.0)	442 (31.8)	181 (23.2)	
Others	2187 (66.0)	639 (56.0)	950 (68.2)	598 (76.8)	
Comorbidities					
Hypertension^a^	1213 (36.7)	267 (23.5)	513 (36.9)	433 (55.6)	< 0.001
Diabetes^a^	470 (14.3)	87 (7.6)	148 (10.7)	235 (30.3)	< 0.001
Dyslipidemia^a^	1554 (47.5)	270 (23.9)	689 (50.3)	595 (76.9)	< 0.001
Cancer^a^	29 (0.9)	11 (1.0)	7 (0.5)	11 (1.4)	0.087
SBP^a^	128.67 ± 20.32	123.30 ± 18.79	128.72 ± 19.60	136.49 ± 21.24	< 0.001
DBP^a^	75.21 ± 11.74	71.96 ± 11.05	75.46 ± 11.45	79.57 ± 11.78	< 0.001
HDL-C	51.03 ± 15.04	58.13 ± 15.93	50.04 ± 12.85	42.40 ± 12.08	< 0.001
LDL-C^a^	116.97 ± 34.44	110.36 ± 31.07	121.28 ± 33.17	118.98 ± 39.60	< 0.001
TC	194.05 ± 38.49	183.68 ± 35.33	195.50 ± 35.95	206.63 ± 42.95	< 0.001
TG	103.54 [73.46, 149.79]	73.46 [58.41, 95.58]	107.08 [81.42, 145.14]	166.38 [119.92, 247.80]	< 0.001
HbA1c^a^	5.28 ± 0.84	5.12 ± 0.57	5.20 ± 0.66	5.66 ± 1.25	< 0.001
TyG2012	8.67 ± 0.66	8.25 ± 0.44	8.66 ± 0.51	9.28 ± 0.71	< 0.001
TyG2015	8.67 ± 0.62	8.24 ± 0.39	8.69 ± 0.48	9.27 ± 0.59	< 0.001
WHtR2012	0.54 ± 0.06	0.48 ± 0.03	0.55 ± 0.04	0.62 ± 0.04	< 0.001
WHtR2015	0.55 ± 0.07	0.48 ± 0.04	0.56 ± 0.04	0.62 ± 0.05	< 0.001
Cumulative TyG-WHtR	14.19 ± 2.19	11.90 ± 0.82	14.36 ± 0.72	17.24 ± 1.18	< 0.001

*TyG* triglyceride-glucose, *SBP *systolic blood pressure, *DBP *diastolic blood pressure, *HDL-C *high-density lipoprotein cholesterol, *LDL-C *low-density lipoprotein cholesterol, *TC *total cholesterol, *TG *triglyceride, *HbA1c *glycosylated hemoglobin, *WHtR *waist height ratio;

^a^ Missing data: 5 for gender; 1 for hukou; 6 for smoking status; 9 for hypertension; 17 for diabetes; 37 for dyslipidemia; 11 for cancer; 11 for systolic blood pressure; 11 for diastolic blood pressure; 6 for low-density lipoprotein cholesterol; 9 for glycosylated hemoglobin

### Odds ratios for incident CVD

During follow-up between 2015 and 2020, 623 participants (18.8%) developed CVD.

**Fig. 3:**
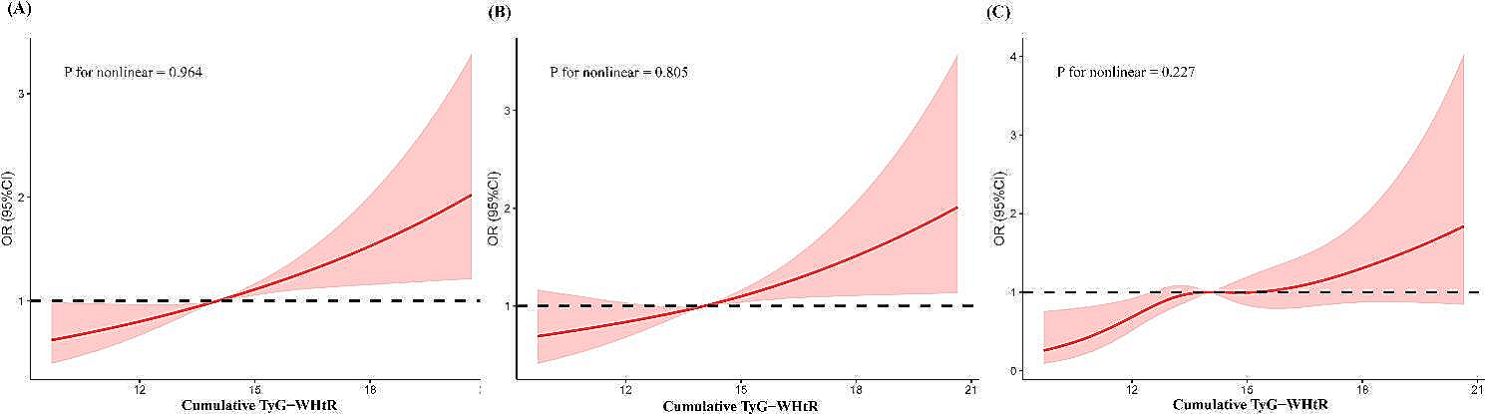
Cubic model of the association between cumulative TyG-WHtR index and (**A**) CVD; (**B**) heart disease;** (**C)stroke

The results of logistic regression analyses for the association between the change of TyG-WHtR and CVD are shown in Table 2. After adjustment for all potential confounders in Model 4, compared with Class 1, the risk of CVD was significantly higher in Class 2 (OR 1.28, 95%CI 1.01–1.63) and Class 3 (OR 1.58, 95%CI 1.16–2.15). The RCS regression model showed a linearly increasing relationship between the cumulative TyG-WHtR and the risk of CVD (P for nonlinearity = 0.964). (Fig. 3) In the fully adjusted model, higher levels of cumulative TyG-WHtR were independently associated with a greater risk of CVD event (per SD, OR 1.27, 95%CI 1.12–1.43). Results were similar when we categorized participants by cumulative TyG-WHtR quartiles. In the final model, compared with the first quartile, the adjusted ORs (95%CI) for CVD were 1.28 (0.96–1.71) for the second quartile, 1.52 (1.13–2.06) for the third quartile, and 1.67 (1.19–2.34) for the fourth quartile. Additional file 1: Table S2 provided the results of logistic regression analyses for heart disease and stroke.

**Table 2:** Logistic regression analysis for the association between different classes of TyG-WHtR and CVD

	Model 1		Model 2		Model 3		Model 4	
	OR(95%CI)	P value	OR(95%CI)	P value	OR(95%CI)	P value	OR(95%CI)	P value
Change in the TyG-WHtR								
1	Reference		Reference		Reference		Reference	
2	1.51 (1.21–1.89)	< 0.001	1.52 (1.21–1.90)	< 0.001	1.33 (1.06–1.68)	0.016	1.28 (1.01–1.63)	0.042
3	2.25 (1.76–2.87)	< 0.001	2.24 (1.75–2.87)	< 0.001	1.68 (1.27–2.23)	< 0.001	1.58 (1.16–2.15)	0.004
Cumulative TyG-WHtR								
1	Reference		Reference		Reference		Reference	
2	1.43 (1.08–1.89)	0.012	1.44 (1.09–1.90)	0.011	1.32 (0.99–1.75)	0.057	1.28 (0.96–1.71)	0.093
3	1.87 (1.42–2.45)	< 0.001	1.88 (1.43–2.48)	< 0.001	1.60 (1.20–2.13)	0.001	1.52 (1.13–2.06)	0.006
4	2.39(1.82–3.14)	< 0.001	2.39(1.81–3.14)	< 0.001	1.77(1.30–2.41)	< 0.001	1.67(1.19–2.34)	0.003
P for trend		< 0.001		< 0.001		< 0.001		0.003
Per SD	1.41(1.29–1.55)	< 0.001	1.41(1.28–1.54)	< 0.001	1.27(1.14–1.42)	< 0.001	1.27(1.12–1.43)	< 0.001

*TyG-WHtR* triglyceride glucose-waist height ratio, *CVD *cardiovascular diseases

**Fig. 4:**
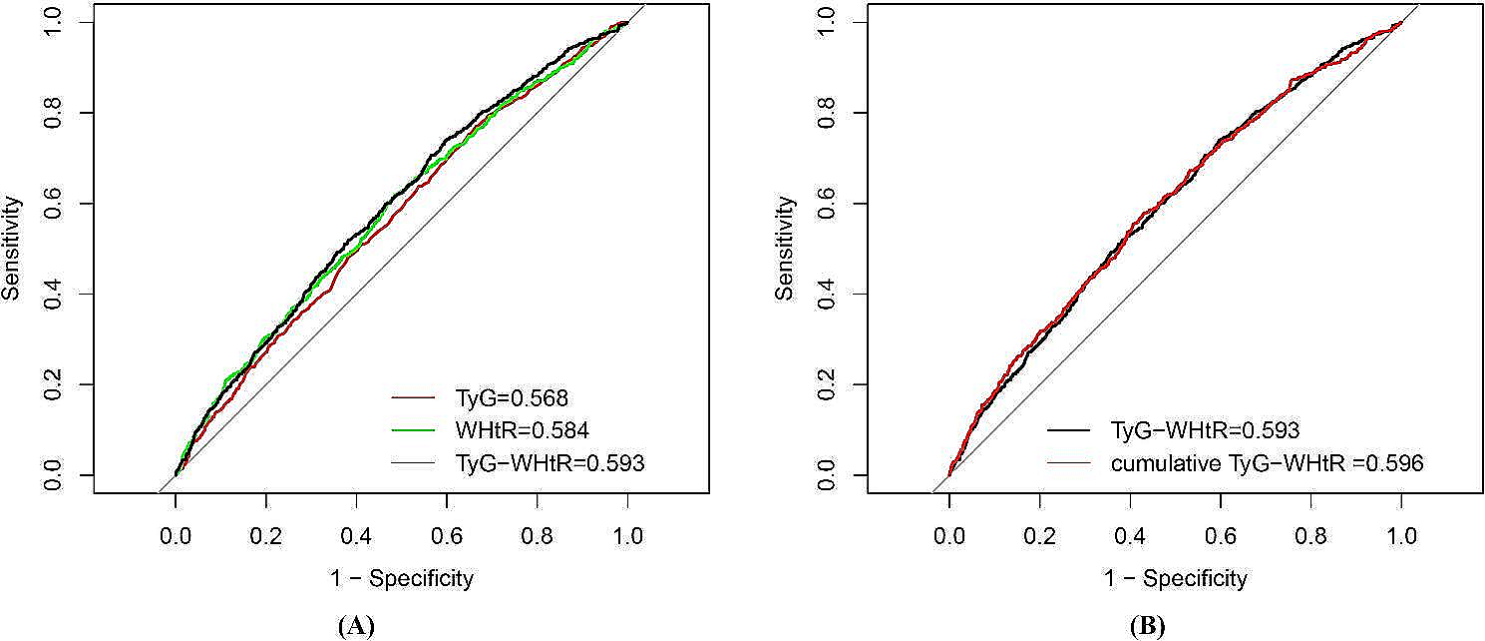
Receiver operating characteristic curves for baseline TyG, WHtR, and TyG-WHtR predicting CVD (**A**) and cumulative TyG-WHtR predicting CVD (**B**)

The ROC curve indicated that cumulative TyG-WHtR (AUC 0.596, 95%CI 0.572–0.621) had the highest diagnostic efficacy for CVD, followed by TyG-WHtR at baseline (AUC 0.596, 95%CI 0.572–0.621), WHtR at baseline (AUC 0.584, 95%CI 0.559–0.609), and TyG at baseline (AUC 0.568, 95%CI 0.544–0.593).(Fig. 4) Cumulative TyG-WHtR also had the highest diagnostic efficacy for heart disease and stroke. (Additional file 1: Figures S2, S3).

### Subgroup analyses and sensitivity analyses

Tables 3, 4 show the association of the change of TyG-WHtR with CVD risk and cumulative TyG-WHtR with CVD risk stratified by different factors, respectively. Except for marital status, subgroups did not have significant interactions. Sensitivity analyses yielded congruent results when performing complete data analyses. (Additional file 1: Table S3).

**Table 3:** Association of change in TyG-WHtR with the risk of CVD stratified by different factors

Subgroup	Change in the TyG-WHt, OR (95% CI)			P for interaction
	Class 1	Class 2	Class 3	
Gender				0.710
Male	Reference	1.23 (0.88–1.73)	1.67 (1.01–2.76)	
Female	Reference	1.41 (0.98–2.04)	1.66 (1.09–2.53)	
Marital status				0.003
Married	Reference	1.30 (1.01–1.68)	1.75 (1.27–2.42)	
Other	Reference	1.16 (0.55–2.43)	0.57 (0.19–1.75)	
Smoking status				0.805
Never	Reference	1.43 (1.02–2.00)	1.76 (1.18–2.63)	
Previous	Reference	1.15 (0.49–2.69)	0.93 (0.28–3.06)	
Current	Reference	1.25 (0.83–1.89)	1.60 (0.86–2.96)	
Drinking status				0.740
Yes	Reference	1.23 (0.83–1.83)	1.29 (0.73–2.29)	
No	Reference	1.32 (0.97–1.79)	1.72 (1.18–2.50)	
Hypertension				0.522
Yes	Reference	1.29 (0.85–1.96)	1.61 (0.99–2.62)	
No	Reference	1.31 (0.97–1.76)	1.49 (0.98–2.26)	
Diabetes				0.806
Yes	Reference	0.95 (0.44–2.07)	0.89 (0.39–2.03)	
No	Reference	1.38 (1.07–1.78)	1.74 (1.24–2.45)	
Dyslipidemia				0.878
Yes	Reference	1.39 (0.93–2.08)	1.74 (1.11–2.73)	
No	Reference	1.17 (0.85–1.61)	1.40 (0.87–2.24)	

*TyG-WHtR* triglyceride glucose-waist height ratio, * CVD *cardiovascular diseases

**Table 4:** Association of cumulative TyG-WHtR with the risk of CVD stratified by different factors

Subgroup	Cumulative TyG-WHt, OR (95% CI)				P for interaction
	Quartile 1	Quartile 2	Quartile 3	Quartile 4	
Gender					0.410
Male	Reference	1.50 (1.03–2.19)	1.49 (0.97–2.30)	1.86 (1.10–3.14)	
Female	Reference	1.08 (0.68–1.73)	1.58 (1.01–2.49)	1.59 (0.98–2.56)	
Marital status					0.006
Married	Reference	1.24 (0.91–1.69)	1.54 (1.12–2.11)	1.82(1.28–2.60)	
Other	Reference	1.68 (0.70–4.02)	1.44 (0.55–3.76)	0.67(0.20–2.23)	
Smoking status					0.605
Never	Reference	1.22 (0.80–1.87)	1.73 (1.13–2.63)	1.83 (1.16–2.88)	
Previous	Reference	1.78 (0.67–4.74)	1.44 (0.50–4.13)	1.09 (0.31–3.84)	
Current	Reference	1.50 (0.95–2.35)	1.45 (0.85–2.46)	1.76 (0.92–3.37)	
Drinking status					0.606
Yes	Reference	1.43 (0.89–2.27)	1.75 (1.07–2.89)	1.48 (0.80–2.74)	
No	Reference	1.21 (0.83–1.75)	1.42 (0.97–2.07)	1.71 (1.13–2.59)	
Hypertension					0.348
Yes	Reference	1.42 (0.84–2.40)	1.40 (0.83–2.37)	1.74 (0.99–3.05)	
No	Reference	1.26 (0.88–1.79)	1.68 (1.16–2.44)	1.57 (1.01–2.45)	
Diabetes					0.652
Yes	Reference	0.73 (0.26–2.04)	1.13 (0.45–2.84)	1.01 (0.40–2.53)	
No	Reference	1.40 (1.03–1.90)	1.65 (1.20–2.28)	1.80 (1.24–2.62)	
Dyslipidemia					0.904
Yes	Reference	1.33 (0.79–2.26)	1.71 (1.03–2.83)	1.88 (1.11–3.20)	
No	Reference	1.28 (0.89–1.83)	1.36 (0.91–2.05)	1.46 (0.89–2.41)	

*TyG-WHtR *triglyceride glucose-waist height ratio, *CVD *cardiovascular diseases

## Discussion

In this study, we observed the relationship between TyG-WHtR changes and CVD. A chronically higher TyG-WHtR or a higher cumulative TyG-WHtR is associated with a higher incidence of CVD events. The ability of TyG-WHtR to assess CVD risk is superior to that of TyG alone and WHtR alone, and cumulative TyG-WHtR, an indicator of dynamic change of TyG-WHtR, has the best performance. However, TyG-WHtR may not assess CVD risks in unmarried population.

Initially, the TyG index alone was used to predict CVD events. In 2016, a groundbreaking study revealed a significant positive correlation between the TyG index and CVD events [6]. Their analysis, which involved 5014 patients from the Vascular Metabolic CUN cohort, spanned a median follow-up period of 10 years. According to the quintiles of the TyG index, they divided the patients into five groups. The risk of CVD in the group with the highest TyG index was 2.32 times that in the group with the lowest TyG index. They also found that the TyG index can improve the accuracy of Framingham model predicting the occurrence of coronary heart disease [6]. Since obesity and cardiovascular disease are closely related, some studies gradually used the TyG index combined with anthropometric and adiposity indicators to predict CVD events. Several studies reported the role of the TyG-BMI index in predicting the incidence of CVD events [15, 17]. A systematic review and meta-analysis suggested the WHtR index was better than BMI and waist circumference at predicting CVD risk [18]. As a good indicator of central (visceral) adipose tissue and a marker of ‘early health risk’ [19], WHtR was recommended to replace BMI in the evaluation of adiposit [20]. A recent study included 11,937 adults from the National Health and Nutrition Examination Survey (NHANES), and they found that the TyG-WHtR index had the highest predictive power for CVD mortality rather than the TyG index [11]. These results suggested that TyG-WHtR will become a more helpful tool for risk stratification and identifying groups at early risk of cardiovascular disease.

Considering that the TyG index changes dynamically over time, researchers began to study the impact of changes on CVD events. Recently, Cui et al. demonstrated that cumulative TyG index was associated with an increased risk of CVD [7]. Huo et al. also found that changes in the TyG-BMI may help identify individuals at higher risk of stroke [15]. However, few studies have investigated the association between changes in TyG-WHtR and CVD.

Our study used K-means clustering analysis to classify individuals into three groups by the change of TyG-WHtR and cumulative TyG-WHtR. Compared to the first group of participants who have consistently low and stable TyG-WHtR, both the second group with moderate and increasing TyG-WHtR and the third group with high TyG-WHtR and slowly increasing trends have higher risks of CVD. Similarly, results grouped according to cumulative TyG-WHtR quartiles had similar findings. Patients with higher cumulative TyG-WHtR have higher CVD risk.

As a reliable biomarker of IR, the TyG index is consistent with the gold standard, the euglycemic-hyperinsulinemic clamp test, and has superior sensitivity and specificity [5]. Compared with the homeostasis model assessment estimated insulin resistance index, the TyG index performs better in evaluating IR [21]. IR can exacerbate both the initiation and progression of atherosclerosis, and the mechanisms involve systemic factors, particularly dyslipidemia, hypertension, and a pro-inflammatory state [22]. IR is independently associated with elevated cardiovascular risk [23].

The evidence from the UK Biobank demonstrated that WHtR was linearly associated with ischemic CVD, myocardial infarction, and ischemic stroke [24]. Raele et al. suggested that WHtR was positively associated with Coronary Artery Calcium and could be regarded as a promising biomarker for subclinical atherosclerosis [25]. Central adiposity is associated with CVD, and WHtR is a good indicator of assessing central adiposity. Obesity leads to adipose tissue dysfunction, and an imbalance in adipokine levels contributes to a chronic systemic inflammatory response, which is fundamental to the development and progression of cardiovascular complications [26]. Our study also showed that TyG-WHtR had better diagnostic performance than TyG alone and WHtR alone in CVD. The indicator cumulative TyG-WHtR, which takes the time span into consideration, has the highest AUC. Similarly, another study also showed that the utilization of the change in TyG-BMI is valuable in assessing the risk for stroke [15]. These results remind us to pay more attention to the value of dynamic monitoring of CVD risk factors.

To verify whether our findings were applicable in different populations, we performed subgroup analyses. Subgroup analyses were consistent with the major findings except in the subgroups of unmarried individuals. Similarly, Huo et al. found that marital status moderates the association of cumulative TyG-BMI with stroke [15]. A previous meta-analysis, including 34 studies and more than two million participants, found that unmarried status was associated with increased odds of CVD [27]. A large prospective cohort study also demonstrated that living singly was associated with a higher risk of CVD [28]. These results suggest that the unmarried population is associated with a higher cardiovascular risk, and the TyG-WHtR index may not predict high-risk CVD individuals in this group.

Our study also has some limitations. Firstly, our participants were all from the Chinese elderly population, so these findings may not be generalizable to other countries or other age groups. Secondly, while the rate of lost follow-up in this study is acceptable, the occurrence of competing events, such as death due to cardiovascular diseases, may underestimate the relationship between TyG and CVD. Last, in our study, CVD is defined as self-reporting heart disease and stroke, which might lead to a misclassification bias. However, previous studies shown that self-reported was basically consistent with medical records and misreporting was nonsystematic, which means the potential misclassification bias was minor [16, 29, 30].

## Conclusion

In our study, we discovered that changes in TyG-WHtR are associated with a higher incidence of CVD events. For individuals with a persistently high and increasing trend in TyG-WHtR over the long term, measures such as exercising, losing weight, or modifying lifestyle habits should be taken to reduce the risk of CVD.

## Data Availability

The datasets used and/or analysed during the current study are available from the corresponding author on reasonable request.

## Supplementary lnformation

Supplementary Material 1.

## Abbreviations

TyG index: Triglyceride-glucose index
CVD: Cardiovascular diseases
TyG-WHtR: Triglyceride glucose-waist height ratio
CHARLS: China Health and Retirement Longitudinal Study
RCS: Restricted cubic spline
SD: Standard deviation
IQR: Interquartile range
OR: Odds ratio
CI: Confidence interval
SBP: Systolic blood pressure
DBP: Diastolic blood pressure
HDL-C: High-density lipoprotein cholesterol
LDL-C: Low-density lipoprotein cholesterol
TC: Total cholesterol
TG: Triglyceride
HbA1c: Glycosylated hemoglobin
