# Supplementary material for: Association between triglyceride glucose-waist height ratio index and cardiovascular disease in middle-aged and older Chinese individuals: a nationwide cohort study
Source: Cardiovasc Diabetol. 2024 Jul 11;23:247. doi: 10.1186/s12933-024-02336-6 (PMC11241990; doi:10.1186/s12933-024-02336-6)
Supplement: Supplementary file 1 — Supplementary Material 1. [file 12933_2024_2336_MOESM1_ESM.docx]

Figure S1: Elbow method to identify the appropriate number of clusters.


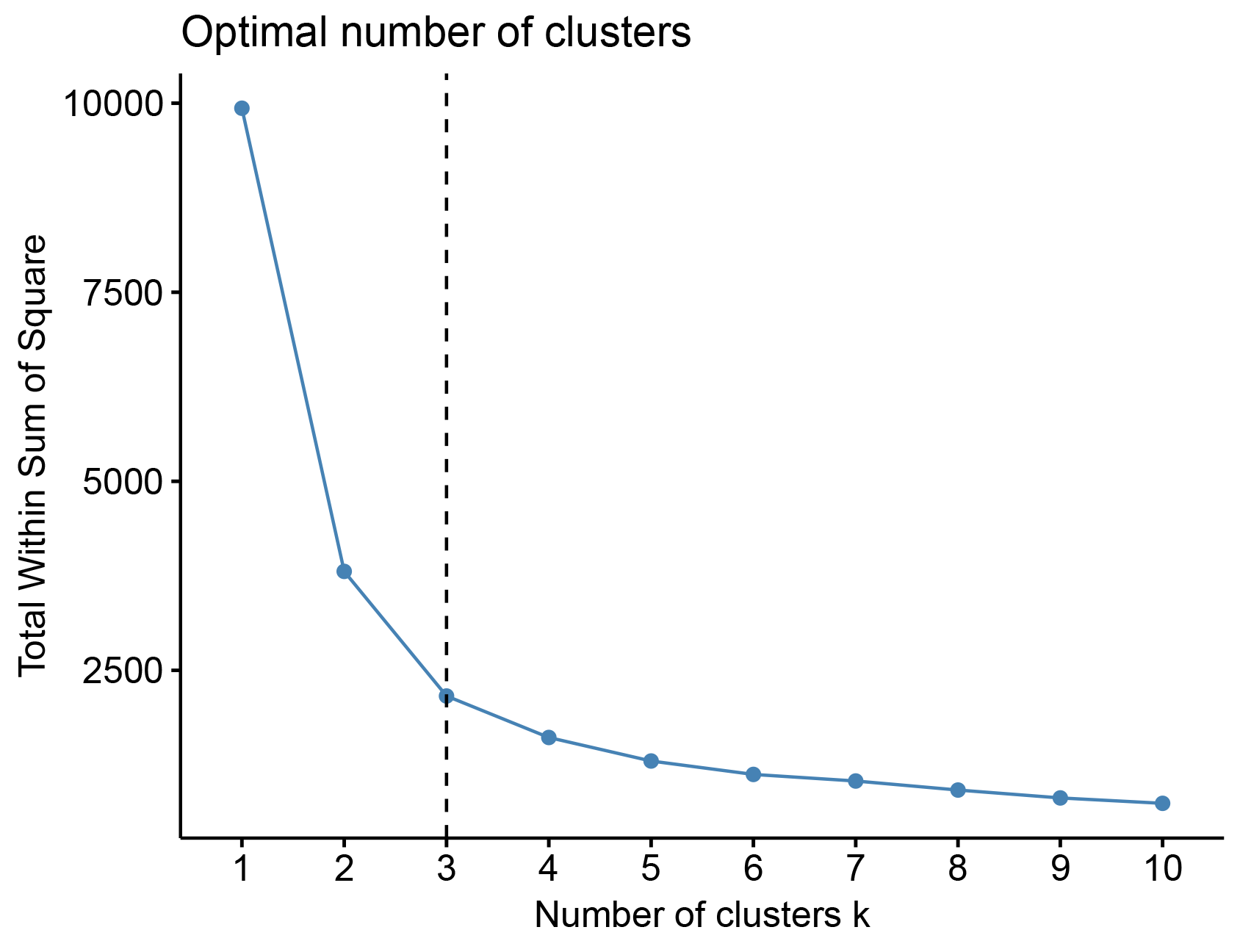


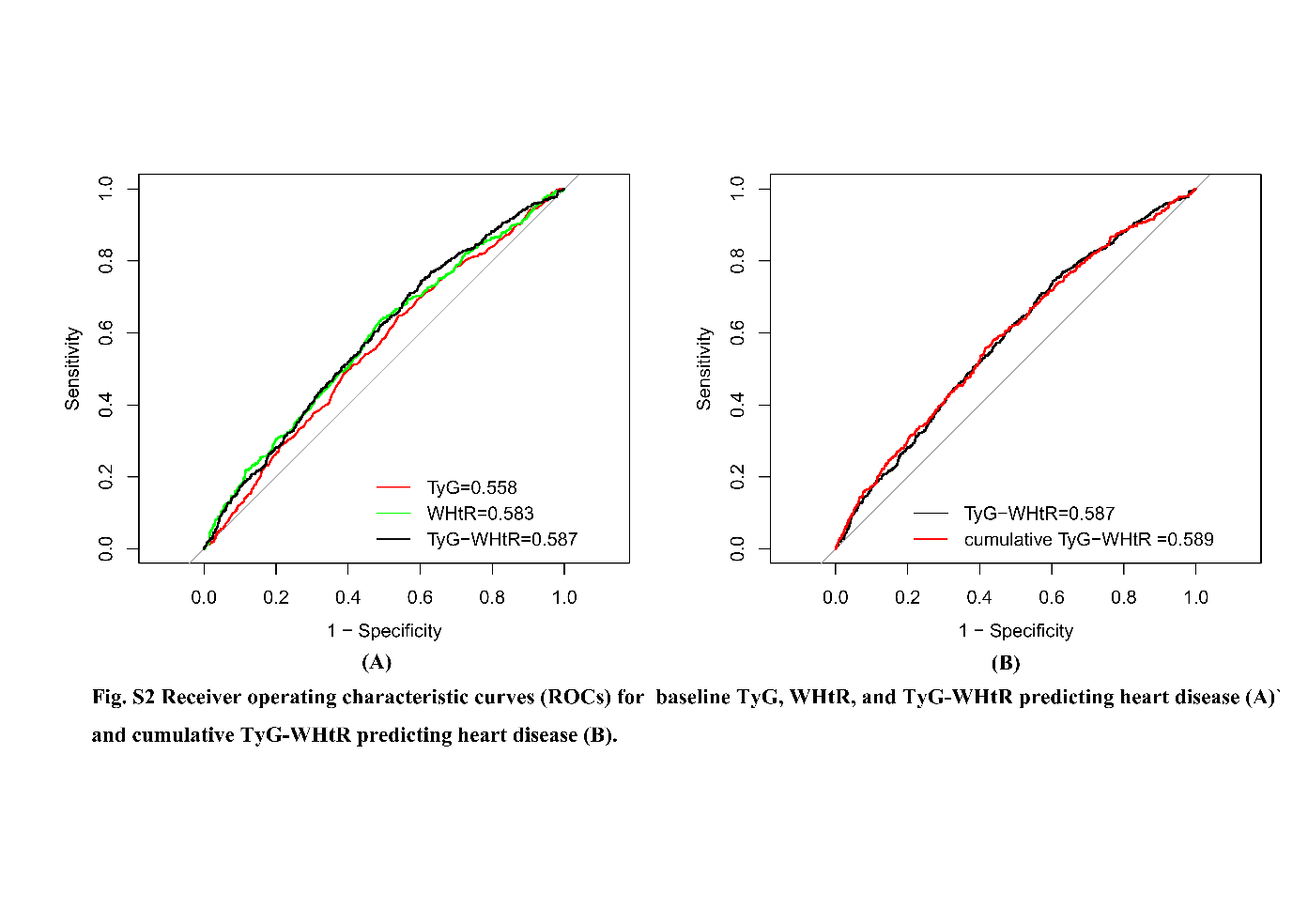





Table S1: Baseline characteristics of participants by cumulative TyG-WHtR quartiles.

| Characteristic | Cumulative TyG-WHtR | | | | P value |
| --- | --- | --- | --- | --- | --- |
|  | Quartile 1 | Quartile 2 | Quartile 3 | Quartile 4 |  |
|  | (n=828) | (n=828) | (n=828) | (n=828) |  |
| Age, years | 58.69±8.51 | 57.94±8.24 | 58.00±8.62 | 58.57±8.10 | 0.153 |
| Gender ^a^ |  |  |  |  |  |
| Male | 566 (68.4) | 406 (49.2) | 319 (38.6) | 208 (25.2) | <0.001 |
| Female | 261 (31.6) | 420 (50.8) | 508 (61.4) | 619 (74.8) |  |
| Marital status |  |  |  |  | 0.978 |
| Married | 746 (90.1) | 742 (89.6) | 746 (90.1) | 747 (90.2) |  |
| Others | 82 (9.9) | 86 (10.4) | 82 (9.9) | 81 (9.8) |  |
| Education |  |  |  |  | <0.001 |
| Lower level | 560 (67.6) | 578 (69.8) | 543 (65.6) | 623 (75.2) |  |
| Higher level | 268 (32.4) | 250 (30.2) | 285 (34.4) | 205 (24.8) |  |
| Hukou ^a^ |  |  |  |  | <0.001 |
| Agriculture | 746 (90.1) | 723 (87.3) | 676 (81.7) | 713 (86.1) |  |
| Others | 82 (9.9) | 105 (12.7) | 151 (18.3) | 115 (13.9) |  |
| Smoking status ^a^ |  |  |  |  | <0.001 |
| Never | 357 (43.2) | 506 (61.1) | 558 (67.6) | 647 (78.3) |  |
| Previous | 67 (8.1) | 57 (6.9) | 73 (8.8) | 44 (5.3) |  |
| Current | 402 (48.7) | 265 (32.0) | 195 (23.6) | 135 (16.3) |  |
| Drinking status |  |  |  |  | <0.001 |
| Current | 371 (44.8) | 306 (37.0) | 255 (30.8) | 193 (23.3) |  |
| Others | 457 (55.2) | 522 (63.0) | 573 (69.2) | 635 (76.7) |  |
| Comorbidities |  |  |  |  |  |
| Hypertension ^a^ | 175 (21.3) | 262 (31.7) | 325 (39.3) | 451 (54.5) | <0.001 |
| Diabetes ^a^ | 61 (7.4) | 67 (8.1) | 99 (12.0) | 243 (29.5) | <0.001 |
| Dyslipidemia ^a^ | 178 (21.7) | 306 (37.5) | 441 (54.0) | 629 (76.5) | <0.001 |
| Cancer ^a^ | 8 (1.0) | 4 (0.5) | 6 (0.7) | 11 (1.3) | 0.294 |
| SBP ^a^ | 122.08 ±17.68 | 126.82 ±19.62 | 129.95 ±20.17 | 135.86 ±21.21 | <0.001 |
| DBP ^a^ | 71.23 ±10.70 | 74.34 ±11.53 | 76.03 ±11.44 | 79.27 ±11.85 | <0.001 |
| HDL | 59.53 ±16.24 | 52.92 ±13.59 | 49.05 ±12.66 | 42.62 ±12.12 | <0.001 |
| LDL ^a^ | 108.63 ±31.04 | 116.82 ±31.63 | 123.81 ±33.44 | 118.66 ±39.36 | <0.001 |
| TC | 182.34 ±35.30 | 189.42 ±35.74 | 198.58 ±35.69 | 205.85 ±42.60 | <0.001 |
| TG | 69.47 [55.76, 91.15] | 93.81 [71.68, 129.21] | 110.62 [84.96, 152.22] | 163.73 [118.59, 246.03] | <0.001 |
| HbA1c ^a^ | 5.11±0.58 | 5.16±0.65 | 5.22±0.65 | 5.64±1.22 | <0.001 |
| TyG2012 | 8.19±0.42 | 8.50±0.47 | 8.71±0.51 | 9.26±0.71 | <0.001 |
| TyG2015 | 8.19±0.37 | 8.49±0.44 | 8.76±0.48 | 9.25±0.59 | <0.001 |
| WHtR2012 | 0.47±0.03 | 0.52±0.03 | 0.56±0.03 | 0.62±0.04 | <0.001 |
| WHtR2015 | 0.47±0.04 | 0.53±0.03 | 0.57±0.03 | 0.62±0.05 | <0.001 |
| Cumulative TyG-WHtR | 11.55±0.69 | 13.31±0.45 | 14.75±0.46 | 17.15±1.20 | <0.001 |

TyG: triglyceride-glucose; SBP: systolic blood pressure; DBP: diastolic blood pressure; HDL-C: high-density lipoprotein cholesterol; LDL-C: low-density lipoprotein cholesterol; TC: total cholesterol; TG: triglyceride; HbA1c: glycosylated hemoglobin; HDL-C; WHtR: waist height ratio;

^a^ missing data: 5 for gender; 1 for hukou; 6 for smoking status; 9 for hypertension; 17 for diabetes; 37 for dyslipidemia; 11 for cancer; 11 for systolic blood pressure; 11 for diastolic blood pressure; 6 for low-density lipoprotein cholesterol; 9 for glycosylated hemoglobin

Table S2. Logistic regression analysis for the association between different classes and heart disease and stroke.

|  | **Heart disease** | | **Stroke** | |
| --- | --- | --- | --- | --- |
|  | OR(95%CI) | P value | OR(95%CI) | P value |
| Change in the TyG-WHtR | |  |  |  |
| 1 | Reference |  | Reference |  |
| 2 | 1.27(0.96-1.67) | 0.089 | 1.20(0.83-1.74) | 0.345 |
| 3 | 1.61(1.13-2.29) | 0.008 | 1.46(0.91-2.33) | 0.114 |
| Cumulative TyG-WHtR | |  |  |  |
| 1 | Reference |  | Reference |  |
| 2 | 1.21(0.87-1.69) | 0.255 | 1.72(1.09-2.72) | 0.021 |
| 3 | 1.45(1.03-2.05) | 0.033 | 1.79(1.10-2.89) | 0.018 |
| 4 | 1.66(1.13-2.43) | 0.010 | 1.94(1.14-3.31) | 0.015 |
| P for trend |  | 0.007 |  | 0.037 |
| Per SD | 1.24(1.08-1.43) | 0.002 | 1.28(1.06-1.54) | 0.009 |

TyG-WHtR: triglyceride glucose-waist height ratio

Adjusted for age, gender, marital status, hukou status, educational level, smoking status, drinking status, history of hypertension, diabetes, dyslipidemia, cancer, systolic blood pressure, diastolic blood pressure, total cholesterol, HDL-C, LDL-C, and HbA1c.

Table S3. Logistic regression analysis for the association between different classes and CVD in subpopulations of 3216 participants with complete data.

|  | **Model 1** | | **Model 2** | | **Model 3** | | **Model 4** | |
| --- | --- | --- | --- | --- | --- | --- | --- | --- |
|  | OR(95%CI) | P value | OR(95%CI) | P value | OR(95%CI) | P value | OR(95%CI) | P value |
| Change in the TyG-WHtR | |  |  |  |  |  |  |  |
| 1 | Reference |  | Reference |  | Reference |  | Reference |  |
| 2 | 1.47(1.18-1.84) | 0.001 | 1.47(1.17-1.85) | 0.001 | 1.30(1.03-1.65) | 0.028 | 1.25(0.98-1.60) | 0.073 |
| 3 | 2.22(1.73-2.85) | <0.001 | 2.21(1.72-2.84) | <0.001 | 1.67(1.26-2.23) | <0.001 | 1.56(1.14-2.14) | 0.005 |
| Cumulative TyG-WHtR | |  |  |  |  |  |  |  |
| 1 | Reference |  | Reference |  | Reference |  | Reference |  |
| 2 | 1.45(1.10-1.93) | 0.010 | 1.46(1.10-1.94) | 0.009 | 1.34(1.01-1.79) | 0.045 | 1.30(0.97-1.75) | 0.079 |
| 3 | 1.85(1.40-2.45) | <0.001 | 1.87(1.41-2.48) | <0.001 | 1.60(1.20-2.15) | 0.002 | 1.52(1.12-2.06) | 0.007 |
| 4 | 2.38(1.80-3.14) | <0.001 | 2.37(1.79-3.14) | <0.001 | 1.78(1.30-2.43) | <0.001 | 1.66(1.18-2.34) | 0.004 |
| P for trend |  | <0.001 |  | <0.001 |  | <0.001 |  | 0.004 |
| Per SD | 1.40(1.28-1.54) | <0.001 | 1.40(1.27-1.54) | <0.001 | 1.27(1.14-1.42) | <0.001 | 1.26(1.11-1.43) | <0.001 |

TyG-WHtR: triglyceride glucose-waist height ratio; CVD: cardiovascular diseases
